# Design combinations of evolved phage and antibiotic for antibacterial guided by analyzing the phage resistance of poorly antimicrobial phage

**DOI:** 10.1128/spectrum.00958-23

**Published:** 2023-09-14

**Authors:** Zhenhe Xu, Zihan Ding, Lijia Shi, YuZhen Xie, Yuanxing Zhang, Shuai Sao, Qiyao Wang, Qin Liu

**Affiliations:** 1 State Key Laboratory of Bioreactor Engineering, Shanghai Frontiers Science Center of Optogenetic Techniques for Cell Metabolism, East China University of Science and Technology, Shanghai, China; 2 Southern Marine Science and Engineering Guangdong Laboratory (Zhuhai), Zhuhai, China; 3 Shanghai Engineering Research Center of Maricultured Animal Vaccines, Shanghai, China; Yangzhou University, Yangzhou, Jiangsu, China

**Keywords:** antibacterial, antibiotics sensitivity, phage evolution, phage therapy, phage resistant, *Pseudomonas plecoglossicida*

## Abstract

Although antibiotics are the primary method against bacterial infections, the rapid emergence of antibiotic resistance has forced interest in alternative antimicrobial strategies. Phage has been considered a new biological antimicrobial agent due to its high effectiveness in treating bacterial infections. However, the applications of phage therapy have been limited by the quick development of phage-resistant bacteria. Therefore, more effective phage treatment strategies need to be explored guided by characterizing phage-resistant mutants. In this study, *Pseudomonas plecoglossicida* phage vB_PpS_SYP was isolated from the sewage but exhibited weak antibacterial activity caused by phage-resistant bacteria. Phage-resistant mutants were isolated and their whole genomes were analyzed for differences. The results showed that mutations in glycosyltransferase family 1 (*GT-1*) and hypothetical outer membrane protein (*homP*) led to bacterial phage resistance. The *GT-1* mutants had lower biofilm biomass and higher antibiotic sensitivity than wild-type strain. Phage SYP evolved a broader host range and improved antimicrobial efficacy to infect *homP* mutants. Therefore, we designed a strategy for combined antibiotic and evolved phage inhibition driven by the two phage-resistant mutants. The results showed that the combination was more effective against bacteria than either antibiotics or phage alone. Our findings presented a novel approach to utilizing poorly antimicrobial phages by characterizing their phage-resistant mutants, with the potential to be expanded to include phage therapy for a variety of pathogens.

**IMPORTANCE:**

The rapid emergence of antibiotic resistance renews interest in phage therapy. However, the lack of efficient phages against bacteria and the emergence of phage resistance impaired the efficiency of phage therapy. In this study, the isolated *Pseudomonas plecoglossicida* phage exhibited poor antibacterial capacity and was not available for phage therapy. Analysis of phage-resistant mutants guided the design of antibacterial strategies for the combination of antibiotics with evolved phages. The combination has a good antibacterial effect compared to the original phage. Our findings facilitate ideas for the development of antimicrobial-incapable phage, which have the potential to be applied to the phage treatment of other pathogens.

## INTRODUCTION

Multidrug resistance (MDR) bacteria are widely recognized as a major health concern. It is estimated that 700,000 people die from MDR globally each year and this number is expected to continue to increase (1, 2). In the post-antibiotic era, phage therapy is frequently considered a possible solution for the treatment of bacterial infections (3, 4). Bacteriophages are bacterial viruses first discovered by Felix d’Herelle and Frederick W. Twort in 1915 (5). Due to the advantages of high specificity, biofilm clearance, self-proliferation, and few side effects, phages are gradually being used in the treatment of MDR bacterial infectious diseases (6 – 8). In recent years, many phages of MDR bacteria, such as *Escherichia coli*, *Salmonella enterica, Klebsiella pneumoniae,* etc., are isolated and have good antibacterial and therapeutic effects on humans and livestock (9 – 12). Phages are classified as emergency investigational new drugs (eINDs) by the Food and Drug Administration (FDA) for the inhibition of MDR bacteria (13). Therefore, phages have great potential to become novel biological antibacterial agents and be applied in clinical treatment.

A major concern for phage therapy is the easy evolution of bacteria to resist phages, which may significantly reduce the efficacy of phage therapy (14). Phage adsorption to bacteria is the first step in infection, and mutations in bacterial surface receptors are a common way to resist phages (15). Typical phage receptors on the surface of bacteria include lipopolysaccharides (LPS), outer membrane proteins (OMP), pili, flagella, etc (16, 17). To resist phages, bacterial mutations are accompanied by a reduction in their fitness, such as growth rate, virulence, biofilm, antibiotic resistance, motility, etc (18 – 20). Altamirano et al. reported that loss-of-function mutations in capsular polysaccharides synthesis genes not only led to resistance of *Acinetobacter baumannii* AB900 to phage øFG02 but also to increased sensitivity to ceftazidime (21). In clinical practice, the combination of antibiotics and phages has a positive effect in reducing bacterial load and improving disease (22). To sum up, although phages can be resisted by mutant bacteria, the reduced fitness of bacteria seems to be beneficial for treatment of infectious diseases.

In microbial ecology, bacteria mutate to resist phages, while phages evolve to infect different bacteria, which are predator-prey dynamics (23). The interaction and co-evolution of bacteria and phages are important causes of microbial diversity, described as the equal opportunity model and royal family model (24). Because of the high evolutionary potential of phages to infect various bacteria, researchers are attempting to drive phage evolution in the laboratory. Borin et al. reported that the trained λ phages, co-cultured with bacteria for 28 days, suppressed bacteria for three to eight times longer than its untrained ancestor, showing a stronger antibacterial effect. Meanwhile, mutation of trained phage resistance is more costly than mutation of untrained phage resistance (25). In summary, phages probably enjoy an advantage in slowing down the evolution of bacterial resistance when co-cultured with bacteria for a few days.

*Pseudomonas plecoglossicida* (formerly named *Pseudomonas putida*) is a common aquatic pathogen that causes visceral granulomas disease in various fish such as *Larimichthys crocea*, *Oncorhynchus mykiss*, and *Epinephelus coioides* (26 – 28). Fish with visceral granulomas disease develop white nodules in the liver, kidneys, and spleen and causing high mortality (27). In this study, phage therapy was desired to control and prevent *P. plecoglossicida*, but newly isolated phage vB_PpS_SYP had poor antimicrobial efficacy. Genomic analysis of phage-resistant mutants revealed that the *GT-1* mutant had increased sensitivity to antibiotics, while the *homP* mutant was well suppressed by the evolved phage. As a result, we designed an antimicrobial strategy combining antibiotics and evolved phages in order to slow the development of phage resistance. Our results suggested a novel idea for the use of poorly antibacterial phages by analyzing the characteristics of phage-resistant bacteria, which contributed to phage therapy for infectious diseases.

## RESULTS

### Isolation of a novel *P. plecoglossicida* phage but with poor antibacterial activity

**Fig 1:**
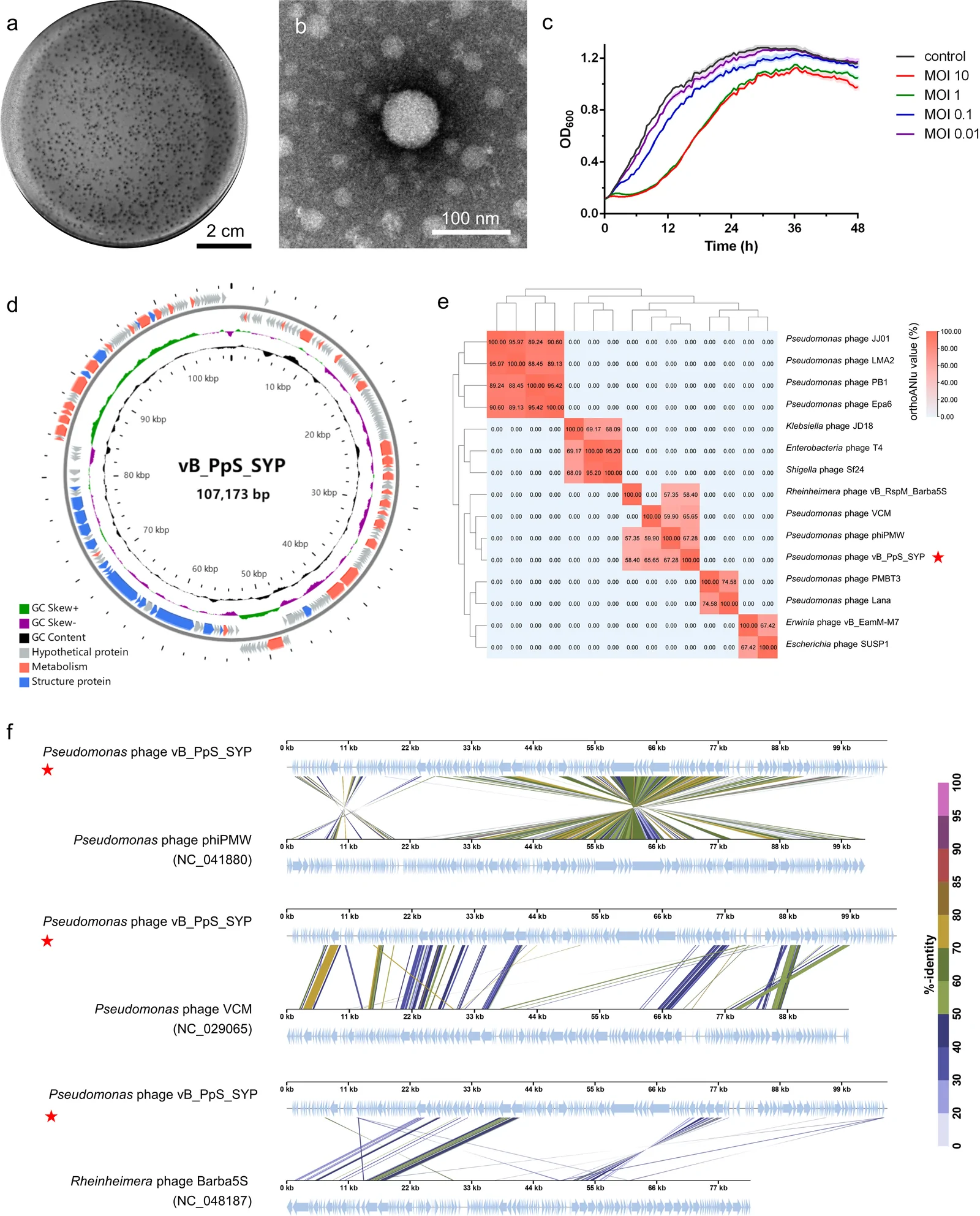
Isolation and characterization of *P. plecoglossicida* phage vB_PpS_SYP. (a) The Plaque morphology on the double-layer agar plate. Scale bar, 2 cm. (b) Transmission electron microscope image. Scale bar, 100 nm. (**c**) Growth curves of *P. plecoglossicida* and phage SYP at multiplicity of infection (MOI) of 10, 1, 0.1, and 0.01 during 48 hours. Bacteria alone served as control. Lines and shaded areas are mean and SD, respectively (*n* = 3). (d) Circle map of the complete genome. CG content is shown in black and GC skew was shown in green and purple. Open reading frames (ORFs) are visualized in the outer circle by arrows in the transcription direction. ORFs are displayed by arrows in the outer circle. (e) The homology between phage genomes was shown in the heatmap. The ANI values, calculated by OrthoANI, for 15 complete phage genomes. The accession numbers of the phages are shown in Table S1. (f) Multiple alignment analysis between *Pseudomonas* phage SYP and *Pseudomonas* phage phiPMW (NC_041880), *Pseudomonas* phage VCM (NC_029065), and *Rheinheimera* phage vB_RspM_Barba5S (NC_048187). The identity percentages are shown in different colors.

The *P. plecoglossicida* phage vB_PpS_SYP (SYP) was isolated from the sewage in Shanghai using *P. plecoglossicida* XSDHY-P as host (Table 1). The phage SYP formed clear plaques of 0.98 ± 0.23mm in diameter on the double-layer agar plate (Fig. 1a). Observed by transmission electron microscope (TEM), phage SYP belonged to the *Podoviridae* family, order *Caudovirales* (Fig. 1b). One-step growth curve of phage SYP showed a latent period of 20minutes and a burst size of 178 PFU/cell (Fig. S1a). Over 80% of free phages adsorbed to bacteria within 5 minutes for subsequent infection (Fig. S1b). The titers of the phage SYP remained stable at pH 5–11 and 4–50℃ (Fig. S1c and d).

**TABLE 1:** Strains or plasmids used in this study

Strains or plasmids	Descriptions	References
***Escherichia coli***		
SM10 λpir	Host for π requiring plasmids, conjugal donor	Lab collection
DH5α λpir	Host for π requiring plasmids	Stratagene
***Pseudomonas plecoglossicida***		
XSDHY-P	Wild-type strain, Amp^r^	(29)
Δ*GT-1*	XSDHY-P, deletion gene of *GT-1*, Amp^r^	This study
Δ*homP*	XSDHY-P, deletion gene of *hom*, Amp^r^	This study
Δ*MDR2*	XSDHY-P, deletion gene of *homP*, Amp^r^	This study
Δ*tetR*	XSDHY-P, deletion gene of *tetR*, Amp^r^	This study
*GT-1^+^*	Δ*GT-1*, containing pBBR1-BAD::*GT-1*, Gm^r^	This study
homP^+^	Δ*homP*, containing pBBR1-BAD::*homP*, Gm^r^	This study
**Plasmids**		
pDMK	Suicide vector, pir dependent, R6K, SacBR, Kan^r^,Cm^r^	Lab collection
pBBR1-BAD	pBBR1 MCS-5 derivative containing the *lac* promoter, Gm^r^	(30)
pBBR1-BAD::*tolC*	pBBR1-BAD carrying the *tolC*, Gm^r^	This study
pBBR1-BAD::*GT-1*	pBBR1-BAD carrying the *GT-1*, Gm^r^	This study
pBBR1-BAD::*homP*	pBBR1-BAD carrying the *homP*, Gm^r^	This study

The complete genome of phage SYP was sequenced and the circle map was displayed in Fig. 1d. The genome of phage SYP is a linear dsDNA containing 107,173 bp and with a CG content of 43.35%. There were 220 predicted open reading frames (ORFs), consisting of 20 structural and packing protein genes, 35 metabolism genes, and 165 hypothetical protein genes. No antibiotic resistance or virulence genes were detected when searching in the Comprehensive Antibiotic Research Database (CARD) and the Virulence Factor Database (VFDB). Meanwhile, no integrase gene was found in the genome, suggesting that phage SYP was a virulent phage and suitable for phage therapy.

The average nucleotide identity (ANI) values were used to determine the similarities between phage SYP and other phages (Fig. 1e). Phage SYP had high ANI values with *Pseudomonas* phage phiPMW (67.28), *Pseudomonas* phage VCM (65.65), and Rheinheimera phage vB_RspM_Barba5S (58.40), suggesting their high similarity. Multiple genome alignments showed that many genes of *Pseudomonas* phage phiPMW shared approximately 70% identity with phage SYP, while only a few genes in *Pseudomonas* phage VCM and *Rheinheimera* phage vB_RspM_Barba5S had some similarity to phage SYP (Fig. 1f). However, phage SYP had an ANI value of 0 with other phages, including some *Pseudomonas* phages, demonstrating that the newly isolated phage SYP was highly different from these *Pseudomonas* phages. To summarize, a novel *Pseudomonas* phage without antibiotic resistance, virulence, and integrase gene was isolated and it had the potential for phage therapy.

To investigate the antibacterial effect, the phage SYP was infected with *P. plecoglossicida* at the multiplicity of infections (MOIs) of 0.01, 0.1, 1, and 10. The absorbance of the bacteria was monitored for 48 hours. Phage SYP has a little antibacterial effect at MOIs of 0.01 and 0.1, while it inhibited bacterial growth at MOIs of 1 and 10 during the first 6 hours (Fig. 1c). But then, the bacteria started growing again due to the rapid emergence of phage-resistant bacteria, indicating that phage SYP had weak antibacterial activity. Therefore, the analysis of phage-resistant bacteria may contribute to the design of antimicrobial strategies.

### Single nucleotide polymorphism analysis showed mutations in *GT-1* and *homP* causing phage resistance

**Fig 2:**
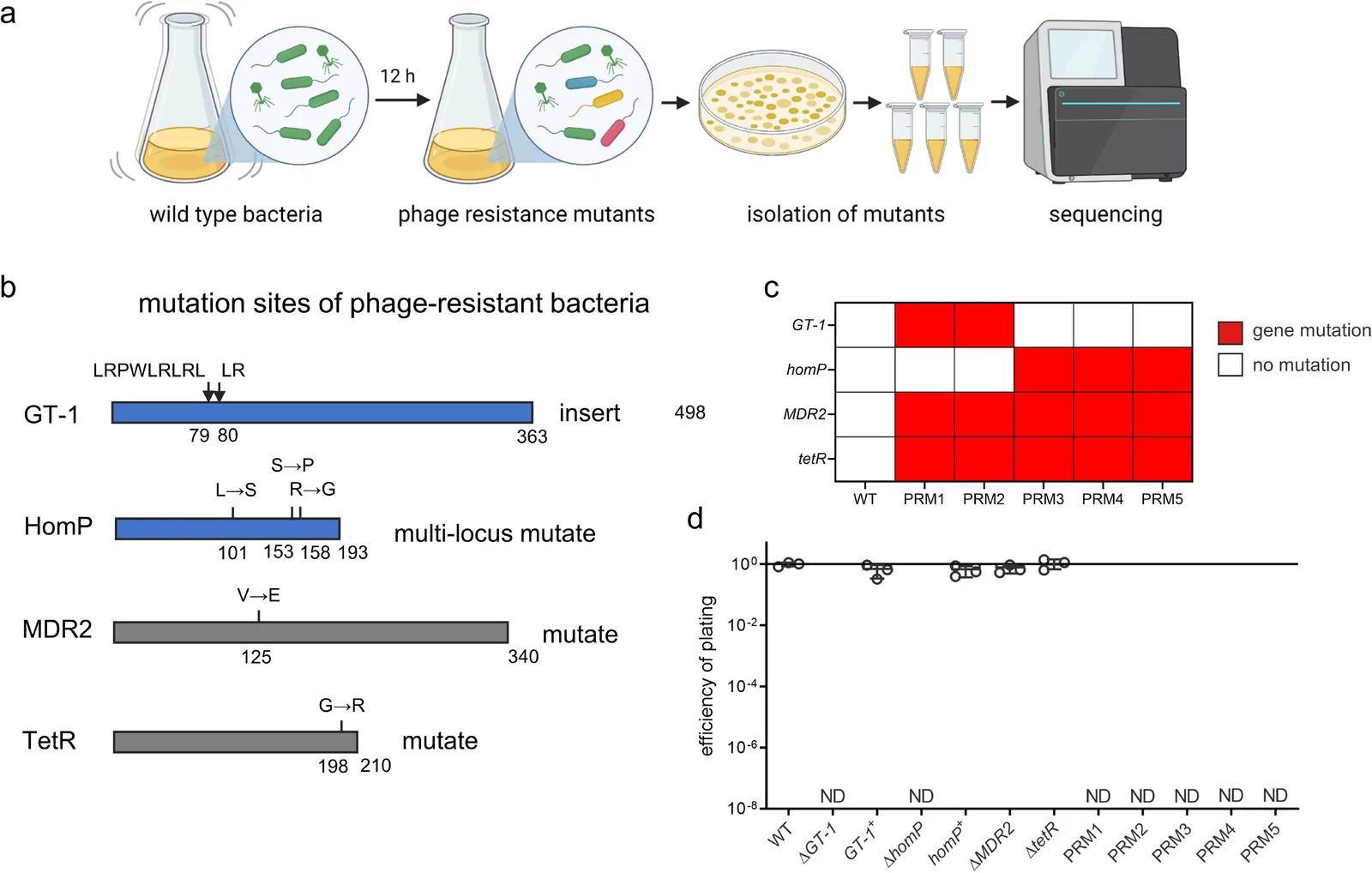
Mutations in *GT-1* and *homP* caused phage resistance. (a) Workflow for isolation of phage-resistant mutants. Phage SYP was co-cultured with the wild-type (WT) strain for 12 hours. Some bacteria mutated to resist the phage and the clones were then isolated on plates. Clones were sequenced to screen for mutated genes. (b) The single nucleotide polymorphisms in phage-resistant mutants. The inserted and mutated amino acids are labeled on the graph. (c) Heatmap of mutated and unmutated genes in five phage resistance mutants. (d) Efficiency of plating (EOP) of phage on wild-type strain, phage-resistant mutants, deletion mutants, and complementary strains. ND means no plaque was detected. The results are shown as the mean ± SD (*n* = 3).

To explore bacterial resistance to phage, phage resistance mutants (PRMs) were isolated on plates and each strain was amplified. Meanwhile, the gene deletion strains and complementary strains were constructed using the prinmers listed in Table 2 to determine the phage resistence genes. Whole genome re-sequencing was used to identify mutant loci in five phage resistance mutants (Fig. 2a). Single nucleotide polymorphism analysis of phage resistance mutants was shown in Table 3. The glycosyltransferase family 1 (GT-1) was inserted by 2 and 9 amino acids in PRM1 and PRM2, respectively. Three different amino acids mutated in the hypothetical outer membrane protein (HomP) in PRM3, PRM4, and PRM5. Both alcohol dehydrogenase (MDR2) and TetR family transcriptional regulator (TetR) also have a single amino acid mutation in each of the mutants (Fig. 2b and c).

**TABLE 2:** Primers used in this study

Primers	Sequence (5'−3')
**Mutants construction**	
GT1-P1	cccccccgagctcaggttacccggatgatacgcccttactcccagc
GT1-P2	gcggttcgccgtatatgtcttcaggatggttcggcgag
GT1-P3	ctcgccgaaccatcctgaagacatatacggcgaaccgc
GT1-P4	gagtacgcgtcactagtggggcccttctagcaggacgccctacatcttcc
GT1-OUT-F	cgccaaagtctacctccttg
GT1-OUT-R	tcgacctgctcaaggaaacc
homP-P1	cccccccgagctcaggttacccggatctattaatcaacgcaccgtcacct
homP-P2	caagatcactaaggttccgtcccgacagaacgaatcatggaagg
homP-P3	ccttccatgattcgttctgtcgggacggaaccttagtgatcttg
homP-P4	gagtacgcgtcactagtggggcccttctagacccgctgaagctctgaatg
homP-OUT-F	cgactcggctgcgaagttg
homP-OUT-R	gacatgtctcgctggagtgctc
MDR2-P1	cccccccgagctcaggttacccggatctatgaaggctccatgctgcgac
MDR2-P2	gacactcgtccatgcagcggaatctgtacctcgccaacg
MDR2-P3	cgttggcgaggtacagattccgctgcatggacgagtgtc
MDR2-P4	gagtacgcgtcactagtggggcccttctaggtcgccaagccatcgagaag
MDR2-OUT-F	gtggaaatcgagtccagtgc
MDR2-OUT-R	ggattacctcaccgcttcagtc
tetR-P1	cccccccgagctcaggttacccggatctatcagcactaccaacctgttcc
tetR-P2	gaacgggccttctacaagccgacagcttcgacctgctg
tetR-P3	cagcaggtcgaagctgtcggcttgtagaaggcccgttc
tetR-P4	gagtacgcgtcactagtggggcccttctaggaaggcttgcgcttgcaag
tetR-OUT-F	cgtgttcatgctgttgaccg
tetR-OUT-R	ggatgatgccattgacctgc
**Complementary strains construction**	
GT1^+^-F	ccatacccgtttttttgggctagcgaattcgggagaggggcactaacccc
GT1^+^-R	ggtcagcatgggtacctttctcctctttaacctgctgacgcgctgagcc
homP^+^-F	ccatacccgtttttttgggctagcgaattcccactcactgggatcgctc
homP^+^-R	ggtcagcatgggtacctttctcctctttaacattgttggtaattgtcgagtcgtgg

**TABLE 3:** SNP analysis of five *P. plecoglossicida* phage resistance mutants

Strain	Position	Reference	Allele	Protein alteration	Annotation
PRM1	274831	T	A	Val125→Glu125	Alcohol dehydrogenase
	2405075	C	T	Gly198→Arg198	TetR family transcriptional regulator
	3113404	-	CAGGCGCAGGCGCAGCCAGGGCCGCAA	363aa→372aa	Glycosyltransferase family 1
PRM2	274831	T	A	Val125→Glu125	Alcohol dehydrogenase
	2405075	C	T	Gly198→Arg198	TetR family transcriptional regulator
	3113401	-	GCGCAG	363aa→365aa	Glycosyltransferase family 1
PRM3	274831	T	A	Val125→Glu125	Alcohol dehydrogenase
	2034405	T	C	Ser153→Pro153	Hypothetical outer membrane protein
	2405075	C	T	Gly198→Arg198	TetR family transcriptional regulator
PRM4	274831	T	A	Val125→Glu125	Alcohol dehydrogenase
	2034250	T	C	Leu101→Ser101	Hypothetical outer membrane protein
	2405075	C	T	Gly198→Arg198	TetR family transcriptional regulator
PRM5	274831	T	A	Val125→Glu125	Alcohol dehydrogenase
	2034420	C	G	Arg158→Gly158	Hypothetical outer membrane protein
	2405075	C	T	Gly198→Arg198	TetR family transcriptional regulator

Gene deletion strains were constructed to examine the efficiency of plating (EOP). Bacteria were not infected by phages SYP when gene *GT-1* and *homP* were knocked out, similar to PRM1–PRM5 (Fig. 2d). When the two genes were complemented, *GT-1* ^+^ and *homP* ^+^ were infected by phage SYP with an EOP similar to that of wild-type (WT) strain. The result showed that mutations in *GT-1* and *homP* promoted bacterial resistance to phage. However, EOP was not significantly decreased when *MDR2* and *tetR* were knocked out, implying that both genes did not contribute to phage resistance. To sum up, gene *GT-1* and *homP* in *P. plecoglossicida* played a key role in bacterial resistance to phage SYP.

### *GT-1* mutations in phage-resistant bacteria increased antibiotic sensitivity

**Fig 3:**
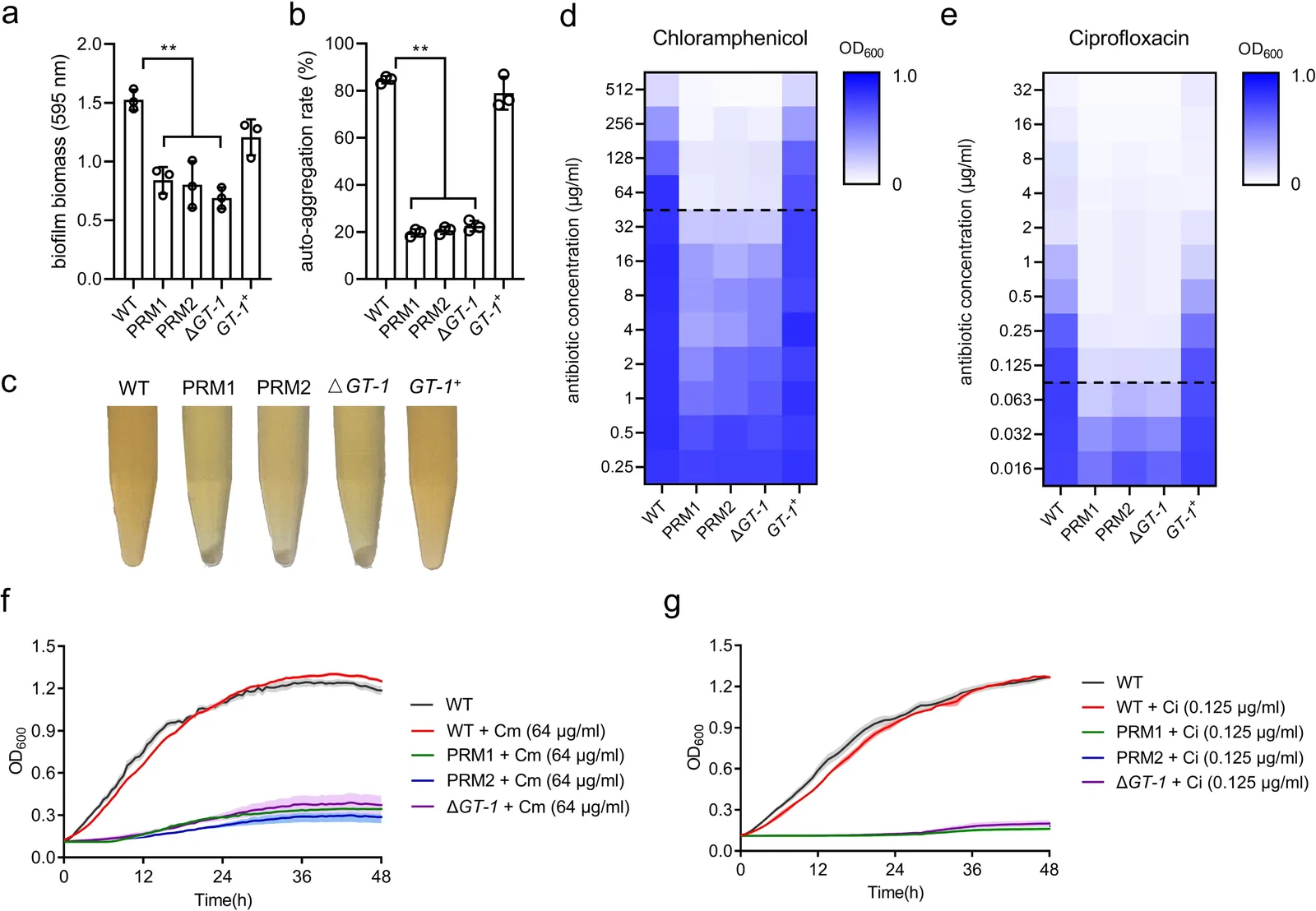
*GT-1* mutants resisted phage SYP but had increased antibiotic sensitivity. (**a**) Biofilm biomass and (**b**) auto-aggregation rate of the mutants. The results are shown as the mean ± SD (*n* = 3). (**c**) Auto-aggregation of mutants after 4 hours of resting. (**d and e**) Increased antibiotic sensitivity of *GT-1* mutants. Optical density at 600 nm of mutants grown in TSB medium containing a twofold gradient dilution of chloramphenicol (**d**) and ciprofloxacin (**e**). OD_600_ ranges from 0 (white) to 1.0 (blue) from three biological replicates. The dashed lines indicate the MIC of *GT-1* mutants. (**f and g**) Growth curves of *GT-1* mutants inhibited by the antibiotic. The mutants were grown in a TSB medium containing 64 µg/mL of chloramphenicol (**f**) or 0.125 µg/mL of ciprofloxacin (**g**). Lines and shaded areas are mean and SD, respectively (*n* = 3).

GT-1 mutations may cause other phenotypic changes in addition to resistance to phage, which may be closely related to their function. The deletion mutant strain and complementary strain were constructed to further explore the properties of phage-resistant bacteria with *GT-1* mutation. The results showed that biofilm biomass generated by the GT-1 mutant bacteria was significantly lower than that produced by the WT strain (Fig. 3a). Meanwhile, the results of the auto-aggregation test showed that the mutant strains were more likely to precipitate compared to the WT strain (Fig. 3). To summarize, the missing glycosyltransferase function of *GT-1* resulted in reduced biofilm biomass and enhanced bacterial auto-aggregation.

Subsequently, MICs of various antibiotics were tested on *GT-1* mutants and WT strains. *GT-1* mutants showed no significant change in sensitivity to ampicillin, kanamycin, tetracycline, streptomycin, and azithromycin, while they showed significantly increased sensitivity to chloramphenicol and ciprofloxacin compared to wild-type bacteria (Fig. S2). WT strains were resistant to chloramphenicol (MIC＞512 µg/mL) but the MIC of chloramphenicol against the *GT-1* mutants was 64 µg/mL (Fig. 3d). In addition, mutations in the *GT-1* gene can reduce the MIC of ciprofloxacin from 2 μg/mL to 0.125 μg/mL (Fig. 3e). Due to reduced MIC, antibiotics were a potential way to inhibit the growth of phage-resistant bacteria. The growth curves of the mutants and WT strains were used to further illustrate the differences in their antibiotic sensitivity. The experimental concentrations of chloramphenicol and ciprofloxacin were 64 µg/mL and 0.125 µg/mL, respectively, which were the MIC for the mutant strain (Fig. 3f and g). WT strains grew well in the medium with chloramphenicol or ciprofloxacin, but the growth of the *GT-1* mutants was significantly inhibited. The results indicated that phage-resistant bacteria increased their antibiotic sensitivity with mutations in the gene *GT-1* and their growth was inhibited by lower concentrations of chloramphenicol and ciprofloxacin. To sum up, the bacteria mutated *GT-1* to resist infection by phage SYP, but the mutant strains had a significantly reduced biofilm and were effectively inhibited by chloramphenicol and ciprofloxacin.

### Evolved phage infected phage-resistant bacteria with a mutation in gene *homP*

**Fig 4:**
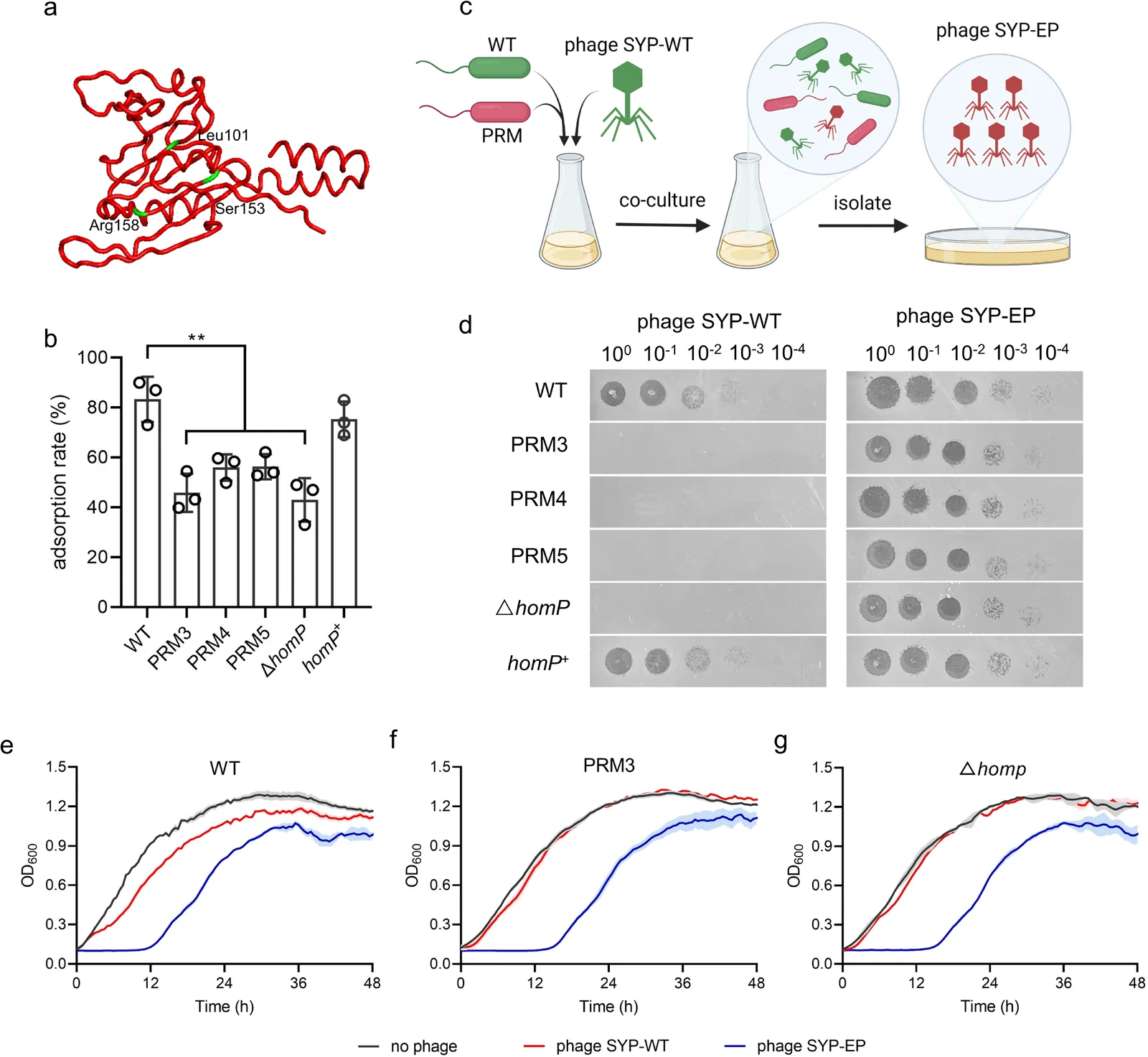
Evolved phage infected phage-resistant bacteria with a mutation in gene *homP*. (**a**) The three-dimensional structure of HomP predicted by RoseTTAFold (confidence: 0.68). Mutated amino acids in phage-resistant mutant strains are marked in green. (**b**) Decreased adsorption of phage SYP-WT to *homP* mutants. The results are shown as the mean ± SD (*n* = 3). (**c**) Workflow of phage evolution to infect phage-resistant bacteria. Phage SYP-WT was co-cultured with wild-type strains and phage-resistant mutants at 28°C for 10 days. Evolved phage (SYP-EP) was isolated on double-layer agar plates with phage-resistant mutants as hosts. (**d**) Phage SYP-WT and SYP-EP were dropped onto the bacterial lawn of wild-type strains, phage-resistant mutants, deletion mutants, and complementary strains. Phages were diluted in a 10-fold gradient. Repeat results may vary with the strength of virus titer. (**e-g**) Antimicrobial effect of phage SYP-EP. Growth curves of wild-type strains (**e**), phage-resistant mutant (**f**), and deletion mutant strain (**g**) in TSB media containing phage SYP-WT or phage SYP-EP. Medium without phage severed as control. Lines and shaded areas are mean and SD, respectively (*n* = 3).

Similar to *GT-1*, mutations in *homP* and its missing function may lead to adverse changes in bacteria. HomP is a hypothetical outer membrane protein in *P. plecoglossicida*. Through the prediction of transmembrane helices, approximately 30 amino acids at the amino-terminal of HomP are transmembrane and the rest of the protein is outside the membrane (Fig. S3a). Additionally, Sec/SPI signal peptide was predicted at the amino-terminal of HomP (Fig. S3b). The protein structure of Homp was predicted by RoseTTAFold with a confidence of 0.68, and three mutant amino acids in PRM were marked in green (Fig. 4a). By scanning electron microscopy (SEM), some white spots were observed on the surface of WT strain and PRM 3 to 5. In contrast, no obvious white spots were evident on the surface of Δ*homP*, presumably, HomP was one of the outer membrane proteins of *P. plecoglossicida* (Fig. S5). When gene *homP* was mutated or knocked out, phage adsorption level to PRM3 to 5 and Δ*homP* was significantly reduced (Fig. 4b). The results suggested that HomP of *P. plecoglossicida* may be a hypothetical outer membrane protein and one of the adsorption receptors for phage SYP.

However, many biological properties were measured to investigate the differences between the *homP* mutant and WT strain but no significant difference was shown. Antibiotics were tried to suppress the *homP* mutants but without significant effect (data not shown). To devise novel strategies to inhibit the growth of *homP* mutants, phage SYP evolved to be infectable with phage-resistant bacteria by co-culture with PRM and WT strains (Fig. 4c). Mutations of evolved phage SYP (phage SYP-EP) occurred in the tail component (gene 137), tail length tape measure protein (gene 142) and a hypothetical protein (gene 126 and 134), mainly in the phage tail structure (Table 4). It was speculated that the changes in the tail structure of phage SYP-EP led to phage adsorption and infection of phage-resistant bacteria with *homP* mutations. In terms of host range, phage SYP-EP formed inhibition zones on plates with *homP* mutations PRM 3 to 5 and Δ*homp*, whereas wild-type phage SYP (phage SYP-WT) failed to inhibit their growth, as tested by dropping plates (Fig. 4d). The growth curves of WT strain and *homP* mutants were measured over 48 hours to examine the antibacterial effect of the evolved phage. Phage SYP-EP completely inhibited the growth of WT bacteria within 12 hours and its antibacterial effect was significantly better than that of the phage SYP-WT (Fig. 4e). Similarly, phage SYP-EP prevented the growth of PRM3 and Δ*homP* within 12 hours, while phage SYP-WT had no antibacterial effect (Fig. 4f and g). In summary, phage SYP-EP was evolved to inhibit the growth of phage-resistant bacteria with *homP* mutations, improving the host range and antimicrobial efficacy.

**TABLE 4:** SNP analysis of *P. plecoglossicida* phage SYP-EP

Gene	Position	Reference	Allele	Protein alteration	Annotation
gene126	52545	G	A	Asp8→Aasn8	Hypothetical protein
gene126	52636	A	G	Lys38→Arg38	Hypothetical protein
gene134	55595	G	A	Ser336→Leu336	Hypothetical protein
gene137	58179	A	C	Ser1264→Ala1264	Tail component
gene137	58295	C	G	Arg1225→Thr1225	Tail component
gene137	58298	A	G	Val1224→Ala1224	Tail component
gene137	58304	A	G	Leu1222→Thr1222	Tail component
gene137	58305	G	T	Leu1222→Thr1222	Tail component
gene137	58380	C	T	Ala1197→Thr1197	Tail component
gene142	66061	T	C	Lys832→Glu832	Tail length tape measure protein
gene142	67208	C	A	Met449→Ile449	Tail length tape measure protein

### Evolved phages have a good antibacterial effect in combination with antibiotics

**Fig 5:**
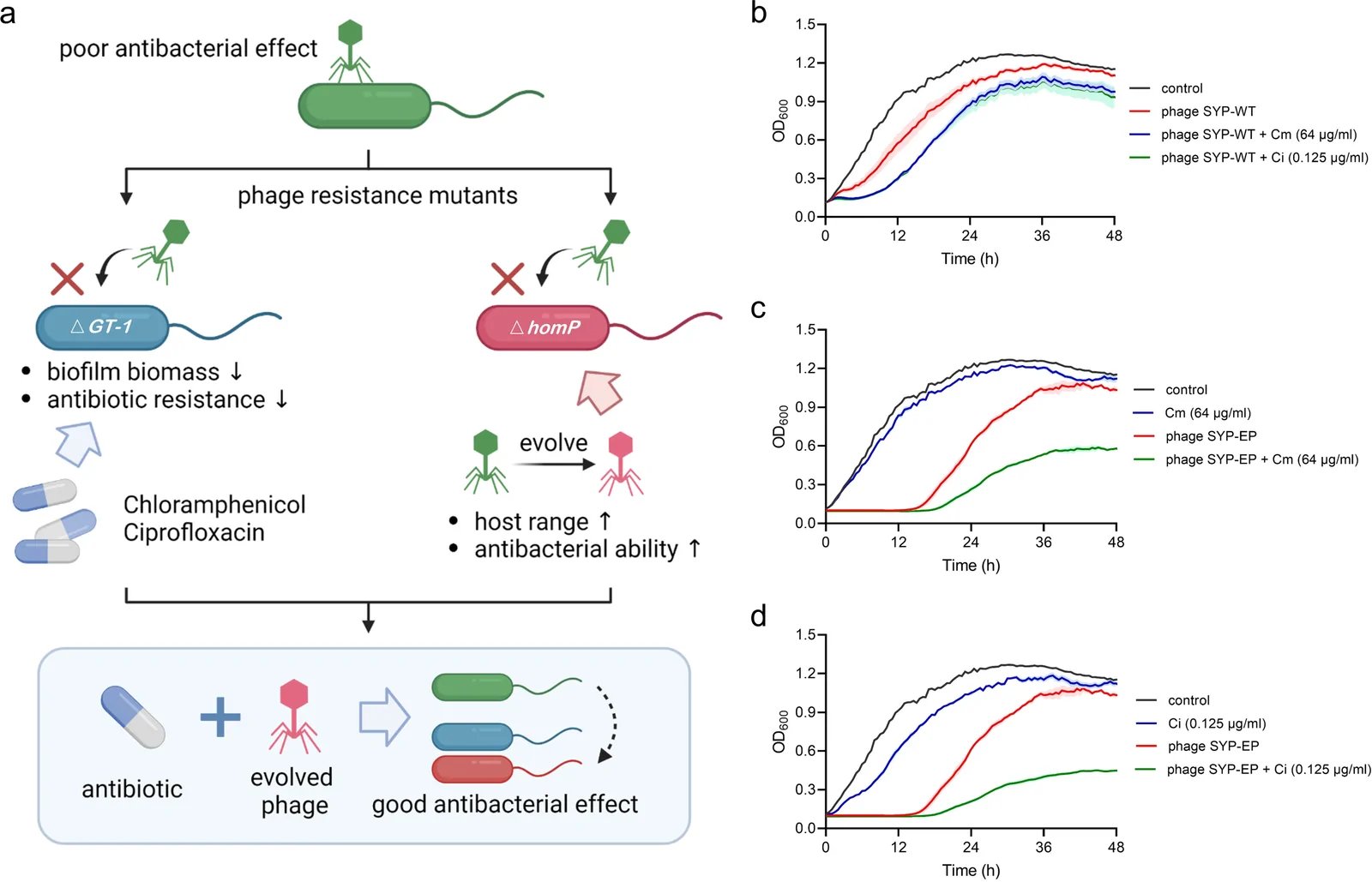
Antibiotics and evolved phages combined to inhibit bacteria growth. (**a**) Design ideas for the combination of antibiotics and evolved phages. The newly isolated phage was poorly antimicrobial due to the rapid emergence of phage-resistant mutants. The genes *GT-1* and *homP* were identified, which were related to phage resistance. The *GT-1* deletion mutant was reduced in biofilm biomass and antibiotic resistance. The *homP* deletion mutant was infected by evolved phages with a wider host range and antimicrobial ability. The combination of antibiotics and evolved phages is designed to inhibit bacteria, resulting in good antibacterial effects. (**b-d**) Growth curves of *P. plecoglossicida* inhibited by the combination of phages and antibiotics. (**b**) Combinations of phage SYP-WT and chloramphenicol or ciprofloxacin. (**c**) Combination of phage SYP-EP and chloramphenicol. (**d**) Combination of phage SYP-EP and ciprofloxacin. Bacteria alone served as a negative control. Chloramphenicol: 64 µg/mL. Ciprofloxacin: 0.125 µg/mL. Lines and shaded areas are mean and SD, respectively (*n* = 3).

Although the phage SYP-WT infected *P. plecoglossicida*, it had a poor antibacterial effect on its own due to the growth of phage-resistant mutants (Fig. S1). Mutations in gene *GT-1* and *homP* of *P. plecoglossicida* caused the phage SYP to fail to infect bacteria. Preventing phage-resistant bacteria in advance may lead to a good antibacterial effect. The *GT-1* mutants showed reduced resistance to chloramphenicol and ciprofloxacin, suggesting that antibiotics were a potential strategy to prevent phage-resistant bacteria. To inhibit *homP* mutants, phage SYP-WT was evolved into phage SYP-EP with improved host range and antibacterial ability. Therefore, we designed a combination strategy using antibiotics and evolved phages to inhibit the emergence of both *GT-1* and *homP* mutants for a stronger antibacterial effect (Fig. 5a).

The growth curves of wild-type bacteria were monitored and compared to examine the antibacterial effect of antibiotic-phage combinations. Although chloramphenicol at 64 µg/mL and ciprofloxacin at 0.125 µg/mL had no antimicrobial effect on wild-type bacteria (Fig. 3f and g), their combination with phage SYP-WT slightly delayed the growth of phage-resistant bacteria and reduced bacterial load (Fig. 5b). For better antibacterial effect, evolved phages were used in combination with antibiotics. Phage SYP-EP, which had stronger antibacterial activity than phage SYP-WT, in combination with chloramphenicol reduced the bacterial load by approximately 60% at 48 hours (Fig. 5c). The combination of ciprofloxacin and phage SYP-EP also showed a significant synergistic antibacterial effect in terms of bacterial load reduction (Fig. 5d). To sum up, the combination of evolved phage SYP-EP with below-MIC chloramphenicol or ciprofloxacin provided better inhibition of *P. plecoglossicida* compared to the poor antimicrobial effect of phage SYP-WT.

## DISCUSSION

Phage therapy is a potential strategy against multidrug-resistant bacteria. It is necessary to obtain different phage strains of pathogens for phage therapy. However, different phages have various growth rates, antimicrobial capabilities, and host ranges, which have a significant impact on the efficacy and application of phage therapy (31). Phage cocktail therapy inhibited bacterial growth well but collecting diverse phage strains was laborious (32). A single phage inhibited bacterial growth for a short time but the bacteria quickly mutated to resist the phage, resulting in a poor antibacterial effect (33). Therefore, the isolation of phages and analysis of their phage resistance mutants are beneficial for phage therapy. In this study, *P. plecoglossicida* phage SYP was isolated but its poor antibacterial effect made it difficult to use for phage therapy (Fig. 1c). Two mutant genes, *GT-1* and *homP*, were identified in phage-resistant bacteria (Fig. 2d). Subsequently, the knockout strains of both genes were characterized to explore bacterial prevention strategies.

GT-1 belongs to glycosyltransferase family 1 (Accession: cl10013), which catalyzes the transfer of sugar moieties and the formation of glycosidic bonds (34). GT-1 affects the synthesis of cellular polysaccharides and polysaccharides on the surface of bacteria are a type of phage adsorption receptors, including lipopolysaccharides (LPS), capsular polysaccharides (CPS), and exopolysaccharides (EPS) (15, 16, 35). Various glycosyltransferases catalyze the synthesis of polysaccharides in bacteria. Mutations in glycosyltransferases caused bacteria to resist phage adsorption and infection. Gong et al. demonstrated that mutations in multiple glycosyltransferases catalyzing LPS and CPS synthesis improved resistance to K1 capsule-specific phage infection in *Escherichia coli* (36). This study reported that mutations in *GT-1*, glycosyltransferase family 1, of *P. plecoglossicida* significantly reduced the bacterial biofilm biomass (Fig. 3A). Moreover, the *GT-1* mutant strains showed significantly reduced resistance to chloramphenicol and ciprofloxacin (Fig. 3d and e). The increased antibiotic sensitivity of phage-resistant mutants was probably caused by a decrease in their biofilm biomass because biofilm formation was a strategy to promote bacterial resistance to antibiotics (37 – 40). However, the phage-resistant mutants had similar MICs to other antibiotics, including ampicillin, kanamycin, tetracycline, streptomycin, and azithromycin, compared to WT strains (Fig. S2). Gao et al. found that different *Salmonella* phage resistance mutants varied in their sensitivity to different antibiotics, similar to our results (41). This was probably because bacteria resist different antibiotics by different mechanisms (42). Alternatively, the surface charge and hydrophobicity of bacteria may vary with the surface structure, thus resisting specific antibiotics (43). In addition, metabolic changes caused by glycosyltransferase deficiency may also affect antibiotic resistance (44). To sum up, chloramphenicol and ciprofloxacin with lower MICs than WT strains were available to inhibit the growth of phage-resistant bacteria with *GT-1* mutations.

HomP was hypothesized to be an outer membrane protein, while the bacterial outer membrane proteins were also important adsorption receptors for phages. Cai et al. revealed that *ompC* of *Klebsiella pneumoniae* was an independent receptor for the phage GH-K3 (45). Some outer membrane proteins of phage receptors had specific functions, such as antibiotic efflux pumps, etc (46). Burmeister et al. reported that phage resistance in *E. coli* via tolC mutations, leading to a reduction in tetracycline and colistin resistance, revealing the trade-off between efflux pumps and phage resistance (47). In this study, phage SYP adsorbed to homP, a hypothetical outer membrane protein, to infect *P. plecoglossicida* (Fig. 2d and 3a). The homP was predicted to be outside the membrane, containing the transmembrane Sec/SPI signaling peptide (Fig. S3). By scanning electron microscopy, homP was observed as a white spot on the cell surface (Fig. S5). Loss of white dots in homP deletion mutants may indicate loss of phage adsorption receptors, leading to phage resistance. Although PRM3 to 5 were resistant to phage SYP, they had white spots on the cell surface. The reason may be that the HomP mutated amino acids to hinder phage adsorption through changes in protein properties without destroying the structure of the protein. In addition, many outer membrane proteins, such as homP, have still not been identified for their functions and need to be further studied. Antibiotics were also considered to inhibit phage-resistant mutants of the *homP* mutation. Unfortunately, *homP* mutants had a similar sensitivity to antibiotics as wild-type bacteria (data not shown). Therefore, other strategies need to be developed to prevent phage-resistant mutants of *homP* mutation.

Several studies described that the co-culture of phages with bacteria allowed for a wider host range of phages and enhanced antibacterial capacity. Habusha et al. reported that *Bacillus subtilis* phage SPO1 evolved to infect resistant bacteria with defects in glycosylated wall teichoic acid (48). Zhang et al. demonstrated that phage evolution trade-off between fast growth rate and wide host range, while only one phage with both advantages was evolved (49). In this study, the phage SYP was co-cultured with the bacteria so that it evolved to infect *homP* mutants (Fig. 4c). The phage SYP-EP had mutations in their tail protein, leading to infection of both wild-type strains and *homP* mutants (Table 4; Fig. 4d). Furthermore, phage SYP-EP inhibited bacterial growth for a longer time compared to phage SYP-WT (Fig. 4e). In short, the phage SYP-EP has been fortunately evolved to have the greater antimicrobial capacity and a wider host range, with great potential for phage therapy. However, phage SYP-EP only inhibited wild-type strain and *homP* mutants for 12 hours, followed by the growth of phage-resistant bacteria. Therefore, there was still a need to explore strategies to extend the duration and improve the effectiveness of antimicrobial activity.

In this study, as antibiotics and evolved phages prevented *GT-1* mutants and *homP* mutants, respectively, the combination of the two was considered to control *P. plecoglossicida* (Fig. 5a). The results showed that the combination of chloramphenicol or ciprofloxacin with phage SYP-EP showed better antimicrobial effect compared to the use of alone (Fig. 5c and d). The enhanced antimicrobial effect was probably due to the advanced control of phage-resistant mutants. In previous studies, antibiotics and phages were often used in combination to treat infectious diseases. Bao et al. reported that the combination of sulfamethoxazole-trimethoprim with the phage cocktail cured a recurrent urinary tract infection with multidrug resistant *Klebsiella pneumoniae* (22). Liu et al. demonstrated that *E. coli* phages had synergistic effects with various antibiotics, driven by a combination of antibacterial mechanism of action and stoichiometry (50). These studies showed that phages synergized with non-sensitive antibiotics. Our study found the same synergy between phages and sensitive antibiotics due to the reduced MIC of phage-resistant bacteria to these antibiotics. Suitable antibiotics can be obtained by screening and used together with phages for treatment. Thus, pre-evolved phage and antibiotic combinations based on the characterization of phage-resistance mutants were effective and potentially extensible approaches. Remarkably, despite the poor antibacterial capacity of the initially isolated phage, we made better use of its therapeutic function through a rational design strategy. This strategy is a possible shortcut in the absence of effective phages to control the pathogens. Furthermore, this strategy can be extended to other pathogens, showing great potential for clinical application. However, only partial phage resistance mutants have increased susceptibility to antibiotics, while phage evolution has been random. Therefore, this strategy needs to be further studied and improved.

Overall, *P. plecoglossicida* phage SYP was isolated and genomically analyzed, but its antimicrobial activity against *P. plecoglossicida* infections was poor. The *GT-1* mutants were resistant to phage SYP infection but had increased sensitivity to chloramphenicol and ciprofloxacin. Phage SYP failed to infect *homP* mutants but can be infected by evolved phage SYP-EP with a wider host range and enhanced antimicrobial activity. Furthermore, the combination of antibiotics and evolved phages produced better antimicrobial effects against *P. plecoglossicida*, compared to alone. In summary, poorly antimicrobial phage was utilized as a strategy for designing antibiotics in combination with evolved phages through analysis of phage resistance mutants. Our work represented a novel phage therapy strategy for the control of *P. plecoglossicida* with a good antibacterial effect, which has great potential for future extension to other pathogens.

## MATERIALS AND METHODS

### Bacterial strains and culture condition

The strains and plasmids used in this study are shown in Table 1. All *P. plecoglossicida* strains were cultured in tryptic soy broth (TSB) medium with shaking at 28°C overnight. *E. coli* strains were cultured in Lysogeny broth (LB) at 37°C. When appropriate, the medium was supplemented with ampicillin (Amp, 100 µg/mL), chloramphenicol (Cm, 25 µg/mL), kanamycin (Kan, 25 µg/mL), or gentamycin (Gm, 30 µg/mL). The 0.05% (wt/vol) L-arabinose was added into cultures to induce P_BAD_ promoter.

### Construction of plasmids, strains, and mutants

*P. plecoglossicida* XSDHY-P was used to construct deletion mutants and complementary strains. The deletion mutants were constructed using sacB-based allelic exchange vectors, as described previously (51). Briefly, the upstream and downstream fragments of the deletions were amplified by PCR. The fragments were ligated to the suicide vector pDMK linearised with *XbaI*. The vectors were transferred from SM10 λpir to *P. plecoglossicida* by conjugation. The single crossover strains were cultured in a TSB medium containing 12% (wt/vol) L-sucrose to screen for the double crossover strains. The deletion mutants were verified by Sanger sequencing. The complement plasmid and strains were constructed, as previously described (30). The plasmid pBBR1 carrying the corresponding gene was transformed into the deletion mutant. All the primers were shown in Table 2.

### Isolation and purification of bacteriophages

The *P. plecoglossicida* phage vB_PpS_SYP was isolated through the double-layer agar plate method (52). Briefly, the sewage was collected from a market in Shanghai and the sample was centrifuged at 8,000 × *g* for 10 minutes. The supernatant was filtered using a 0.22 µm polycarbonate membrane (Millipore, USA). Then, 2 mL of the bacterial culture (OD_600_ = 0.4–0.6) and 100 mL of 2 × TSB were added to 100 mL of the filtrate. The mixture was cultured at 28°C with shaking overnight. The culture was centrifuged at 8,000 × *g* for 5 minutes and the supernatant was filtered through a 0.22 µm polycarbonate membrane. Afterward, the diluted filtrate and bacterial culture (OD_600_ = 0.4–0.6) were added to the TSB medium (at approximately 60°C) containing 0.7% agar. The mixture was poured onto a TSA plate and the plate was cultured at 28°C for 12 hours. The plaque was picked up and placed in the Super Micro buffer (SM buffer: 200 mM NaCl, 16 mM MgSO4, 0.1 M Tris-HCl, 0.02% gelatin, pH 7.5). The process was repeated three times. The phage was concentrated through the NaCl-PEG precipitation method and purified by cesium chloride density gradient ultracentrifugation, as described previously (53).

### Phage characteristics

The morphology of the phage was observed by TEM. The phage was added to carbon-coated copper grids for 5 minutes and the phosphotungstic acid (2%, pH 7.0) was added for 30 seconds. The sample was observed by TEM (JEM-1400plus; JEOL, Japan) at an acceleration voltage of 80 kV. Six images were obtained for each sample.

The one-step growth curve was measured, as described previously, with some modifications (54). The phage was incubated with bacteria at an MOI of 0.1 for 10 minutes and then centrifuged at 12,000 × *g* at 4°C for 2 minutes. The pellet was resuspended with 1 mL TSB and then added to 10 mL TSB. The suspension was cultured with shaking at 28°C and the phage titers were measured every 10 minutes using the double-layer agar method. Each treatment was performed in triplicate. The burst size was calculated by dividing the final phage titer by the initial phage titer.

The adsorption rates of phage to the wild-type strain, phage-resistant mutants, deletion mutants, and complementary strains were measured similarly to previous studies (55). The bacterial culture was diluted to a concentration of approximately 1 × 10^8^ CFU/mL with TSB medium. The phage was added at an MOI of 0.001 and incubated with shaking at 28°C. Samples were taken at 2.5 minutes intervals within 20 min and centrifuged at 8,000 × *g* for 5 minutes. The phage titer of the supernatant was determined as free phage concentrations by the double-layer agar method. The adsorption rate was calculated as follows: adsorption rate (%) = [(initial phage titer – phage titer in the supernatant)/(initial phage titer)] ×100.

The thermal stability and pH stability of the phage were determined according to the previous method, as described previously. Briefly, phages were incubated at different pH levels (pH 3 to 12) and temperatures (4, 16, 28, 37, 50, 60, and 70°C) for 1 hour. The phage titers were measured by the double-layer agar method. Each treatment was performed in triplicate replicates.

### Genome sequencing and bioinformatics analysis

The genomic DNA of the bacteria and phage was extracted using the TIANamp Bacteria DNA Kit and the TIANamp Virus DNA/RNA Kit, respectively (Tiangen, China). Library construction and sequencing were conducted as the previous method (56). The phage genome was sequenced on an Illumina NovaSeq platform and assembled by A5-miseq (57) and SPAdes (58). GeneMark (59) was used to predict ORFs. The protein-coding genes were annotated by searching against the NCBI Nonredundant (NR), VFDB (60), and CARD (61) databases. The phage circular map was generated with CGView (62) and the heatmap was generated with TBTools (63). The OrthoANIu tool was used to calculate the ANI values between two phages (64). The multiple sequence alignment was visualized using the ViPTree server (65). For whole genome re-sequencing, SNPs, and InDel were detected and annotated with GATK (66) and ANNOVAR (67). SNPs were identified at a minimum of 90% variant frequency and minimum coverage of 50. The genomes of *P. plecoglossicida* XSDHY-P (CP031146) and phage SYP (OQ183418) served as reference genomes for mutant bacteria and phages, respectively.

### Antibacterial effect *in vitro*

The bacteria culture was centrifuged and resuspended in phosphate buffered saline (PBS). The phage was concentrated and purified using the NaCl-PEG method. Then, the bacteria (2 µL) and the phage (2 µL) were added to the plates at an MOI of 0.1. TSB media with corresponding antibiotics was supplemented to a final volume of 200 µL. The bacteria treated with PBS served as the negative control. Plates were cultured at 28°C for 48 hours with shaking. The optical density at 600 nm was monitored with a Bioscreen C plate reader (Oy Growth Curves AB Ltd, Finland) every 30 minutes. Each treatment was performed in triplicate.

### Isolation of phage-resistant mutants

Wild-type bacterial cultures and phage SYP were added to the TSB medium at an MOI of 0.1. The mixture was co-cultured with shaking at 28°C for 12 hours and then centrifuged at 8,000 × *g* for 5 minutes. The bacteria were washed three times and graded diluted with PBS. The dilutions were spread on TSA plates and incubated overnight at 28°C. The clones were picked and amplified in TSB medium. The clones were verified to resist phage SYP by drop plate test. The phage-resistant mutants were stored in 40% (vol/vol) glycerol at −80°C for long-term storage.

### Efficiency of plating assays

The EOP was measured, as described previously (68). In brief, phages were diluted in a 10-fold gradient with SM buffer. 2 µL of dilution was spotted on the bacterial lawn and the plates were cultured at 28°C overnight. Phage titers were determined through the number of plaques multiplied by the dilution factor. The wild-type strain served as a control. Each treatment was performed in triplicate. EOP was calculated as follows: EOP = phage titer on target bacteria/phage titer on control bacteria.

### Auto-aggregation and biofilm formation assays

Wild-type strains, phage-resistant mutants, and deletion mutants were cultured in TSB media with shaking at 28°C overnight. Cultures were diluted to a concentration of 3 × 10^9^ CFU/mL using a TSB medium and dispensed into tubes. Tubes were stored at 4°C for 4 hours and then observed for the auto-aggregation of bacteria. The auto-aggregation rate was calculated as follows: auto-aggregation rate(%) = (initial OD_600_ − post-incubation OD_600_)/ initial OD_600_ × 100. For biofilm assays, bacteria were inoculated into a TSB medium in a 96-well plate. The plate was incubated at 28°C for 24 hours. The cell culture was then removed and washed gently three times with PBS. Anhydrous methanol was added to the plate and incubate for 15 minutes. For staining, crystalline violet solution (0.1%) was added to the plate to stain and incubated for 20 minutes. After washing three times with PBS, 95% ethanol was added to the plates and incubated for 1 hour. The optical density at 595 nm was measured using a microplate reader.

### Assessment of antibiotic resistance

Eight antibiotics including ampicillin, azithromycin, chloramphenicol, ciprofloxacin, kanamycin, streptomycin, and tetracycline were used to determine the antibiotic sensitivity of bacteria. The TSB media with different antibiotic concentrations were diluted in a twofold gradient. The bacterial culture (2 µL) and corresponding TSB media were added to the plate. The plate was incubated at 28°C for 48 hours and OD_600_ was measured using a microplate reader. Each treatment was performed in triplicate.

### Scanning electron microscope

The bacteria were cultured on the wafer and washed three times with PBS. Then the wafers were incubated in 2.5% glutaraldehyde for 12 hours and washed with PBS deionized water. Wafers were sequentially immersed in 30%, 50%, 70%, and 100% ethanol to dehydrate for 5 minutes each time. The sample was freeze-dried and then mounted on a holder. After coating with a gold layer, the surface structure of the bacteria was observed by scanning electron microscopy (FEI Volumescope 2, USA).

### Evolution of phages and host range assays

The evolution of the phage is, as described previously, with modifications (69). In brief, the phage SYP-WT was co-cultured with wild-type strains and phage-resistant mutants at 28°C for 24 hours. The mixture was then inoculated into a fresh TSB medium and cultured for 24 hours. The process was repeated for 10 rounds. The evolved phage was isolated on the double-layer plate using phage-resistant mutants. Evolved phage was diluted in a 10-fold gradient with SM buffer. The dilution was dropped onto the bacterial lawn to verify the antibacterial effect. Each treatment was performed in triplicate. The plate of drop tests may vary from different batch of phage fermentation but lead to the same result of host range.

### Statistical analysis

Statistical analyses were performed with GraphPad Prism 7.0. Results were expressed as means ± SD. Unpaired two-tailed Student’s *t*-test was used to determine the statistical significance, and the significance (*P ≤ 0.05 and **P ≤ 0.01).

## Data Availability

The whole genome re-sequencing data were submitted to GenBank under BioProject ID: PRJNA931797 and PRJNA934256. The sequence data for the *Pseudomonas* phage vB_PpS_SYP were deposited at GenBank under accession no. OQ183418.

## SUPPLEMENTAL MATERIAL

Supplemental table and figures spectrum.00958-23-s0001.pdf Table S1 and Fig. S1 to S5.

ASM does not own the copyrights to Supplemental Material that may be linked to, or accessed through, an article. The authors have granted ASM a non-exclusive, world-wide license to publish the Supplemental Material files. Please contact the corresponding author directly for reuse.
