# Supplementary material for: Design combinations of evolved phage and antibiotic for antibacterial guided by analyzing the phage resistance of poorly antimicrobial phage
Source: Microbiol Spectr. 2023 Sep 14;11(5):e00958-23. doi: 10.1128/spectrum.00958-23 (PMC10580904; doi:10.1128/spectrum.00958-23)
Supplement: Supplemental table and figures — Table S1 and Fig. S1 to S5. [file spectrum.00958-23-s0001.pdf]

Supplementary Materials

TABLE S1 The accession numbers of the phages used in this study.

phage strains	accession no.
Enterobacteria phage T4	NC_000866.4
Erwinia phage vB_EamM-M7	HQ728263.1
Escherichia phage SUSP1	NC_028808.2
Klebsiella phage JD18	KT239446.1
Pseudomonas phage phiPMW	NC_041880.1
Pseudomonas phage VCM	NC_029065.1
Rheinheimera phage vB_RspM_Barba5S	NC_048187.1
Shigella phage Sf24	MF327008.1
Pseudomonas phage vB_PpS_SYP	OQ183418.1
Pseudomonas phage PMBT3	MG596799.1
Pseudomonas phage Lana	MK473373.2
Pseudomonas phage PB1	EU716414.1
Pseudomonas phage Epa6	MT108726.1
Pseudomonas phage JJ01	ON324181.1
Pseudomonas phage LMA2	FM201282.1

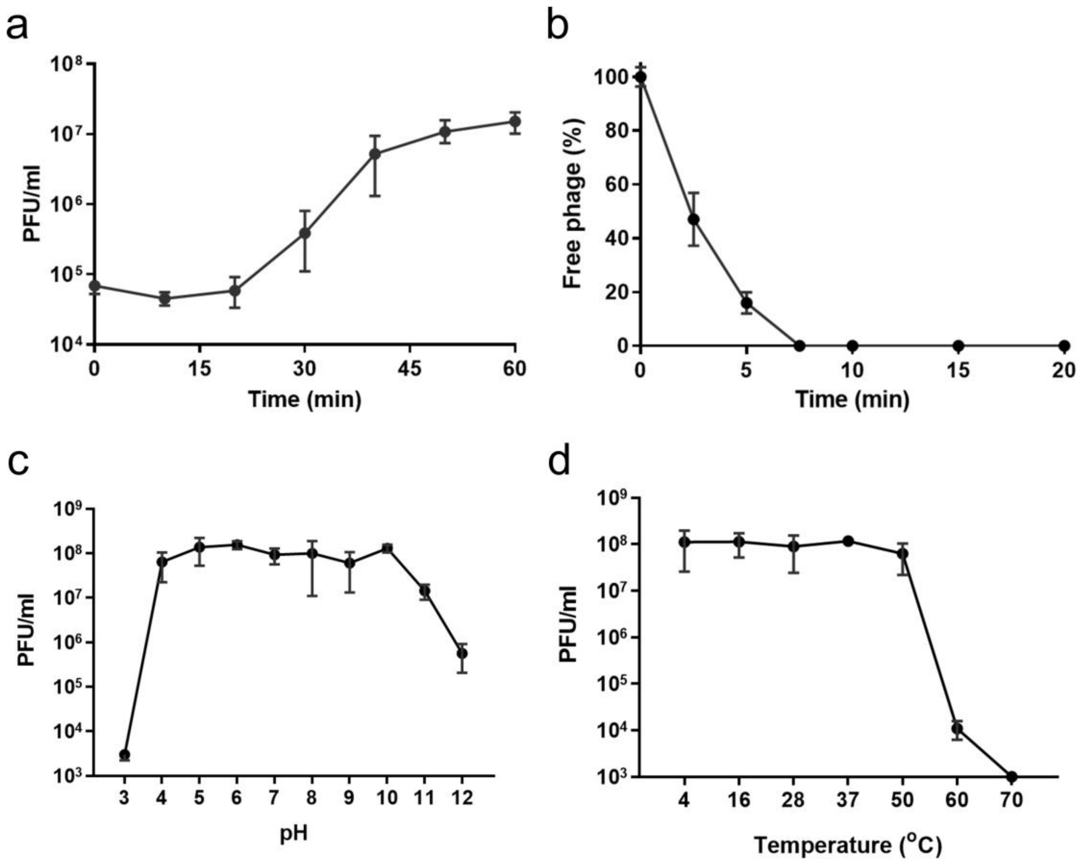

FIG S1 (a) One-step growth curve of phage vB_PpS_SYP. (b) Dynamics of phage SYP adsorption to hosts. (c) pH stability of phage SYP treated with different pH for 2 h. (d) Thermal stability of phage SYP treated with different temperature for 2 h. The results are shown as the mean ± S.D. (*n* = 3).

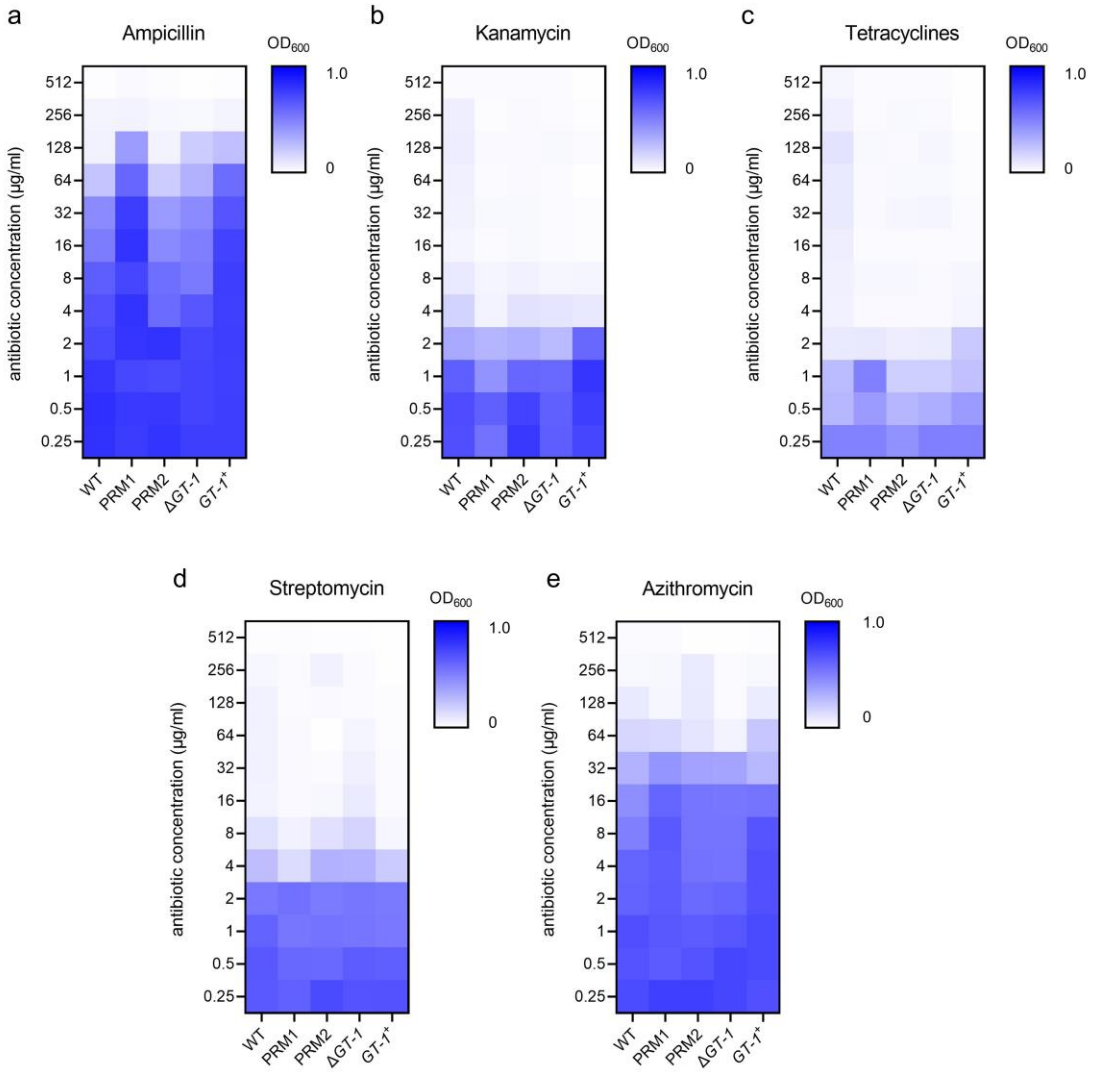

FIG S2 Antibiotic sensitivity of *GT-1* mutants, wild-type strains, and complementary strains. Optical density at 600 nm of mutants grown in TSB medium containing a 2-fold gradient dilution of (a) Ampicillin, (b) Kanamycin, (c) Streptomycin, (d) Tetracycline, or (e) Azithromycin. OD_{600} ranges from 0 (white) to 1.0 (blue) from three biological replicates.

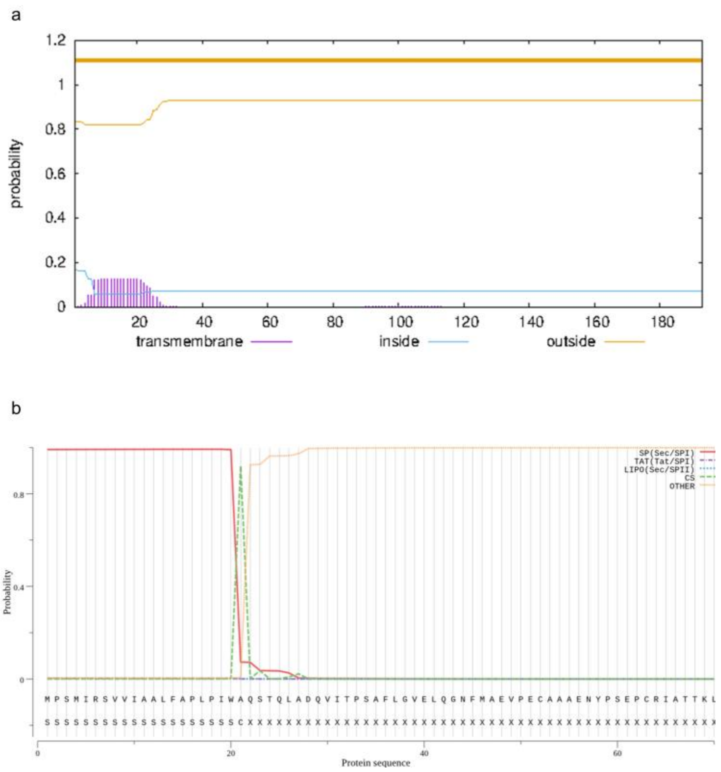

FIG S3 Prediction of Homp characteristics. (a) Transmembrane helices in proteins. (b) Signal peptide and cleavage sites.

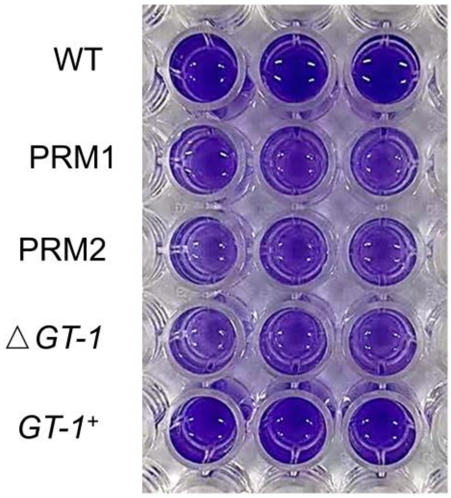

FIG S4 Biofilm of the GT-1 mutant strain and its crystalline violet staining.

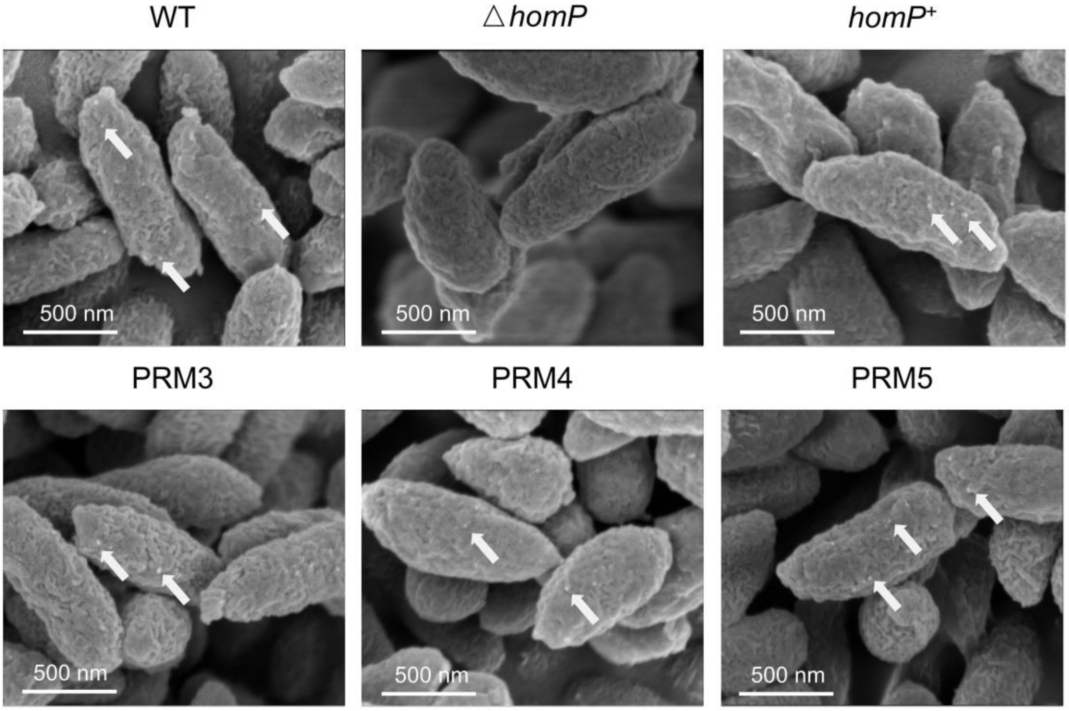

FIG S5 Scanning electron microscope images of the wild-type strain, phage-resistant mutants, deletion mutants, and complementary strains. Arrows point to white dots on the surface of the bacteria. Scale bar, 500 nm.
